# Investigation of Risperidone Treatment Associated With Enhanced Brain Activity in Patients Who Stutter

**DOI:** 10.3389/fnins.2021.598949

**Published:** 2021-02-12

**Authors:** Gerald A. Maguire, Bo Ram Yoo, Shahriar SheikhBahaei

**Affiliations:** ^1^School of Medicine, University of California, Riverside, Riverside, CA, United States; ^2^National Institute of Neurological Disorders and Stroke/National Institutes of Health, Bethesda, MD, United States

**Keywords:** stuttering, risperidone, positron emission tomography, astrocyte, dopamine

## Abstract

Stuttering is a childhood onset fluency disorder that leads to impairment in speech. A randomized, double-blinded placebo-controlled study was conducted with 10 adult subjects to observe the effects of risperidone (a dopamine receptor 2/serotonin receptor 2 antagonist) on brain metabolism, using [^18^F] deoxyglucose as the marker. At baseline and after 6 weeks of taking risperidone (0.5–2.0 mg/day) or a placebo pill, participants were assigned to a solo reading aloud task for 30 min and subsequently underwent a 90-min positron emission tomography scan. Paired *t*-tests were performed to compare the pre-treatment vs. post-treatment in groups. After imaging and analysis, the blind was broken, which revealed an equal number of subjects of those on risperidone and those on placebo. There were no significant differences in the baseline scans taken before medication randomization. However, scans taken after active treatment demonstrated higher glucose uptake in the specific regions of the brain for those in the risperidone treatment group (*p* < 0.05). Risperidone treatment was associated with increased metabolism in the left striatum, which consists of the caudate and putamen, and the Broca’s area. The current study strengthens previous research that suggests the role of elevated dopamine activity and striatal hypometabolism in stuttering. We propose that the mechanism of risperidone’s action in stuttering, in part, involves increased metabolism of striatal astrocytes. We conclude that using neuroimaging techniques to visualize changes in the brain of those who stutter can provide valuable insights into the pathophysiology of the disorder and guide the development of future interventions.

## Introduction

Stuttering is a neurodevelopmental disorder characterized by frequent disruptions during speech, silent blocks, and repetitions or prolongations of sounds and syllables (Maguire et al., 2020). The fifth edition of the American Psychiatric Association Diagnostic and Statistical Manual (DSM-V) defines stuttering as a disturbance in the normal fluency and time patterning of speech that is inappropriate for the individual’s age and language skills (American Psychiatric Association, 2013). Along with repetitions, prolongations, avoidance or anxiety around speaking situations, and physical tension, there may be involuntary motor movements such as tics, tremors, and eye blinks (Maguire et al., 2020). Those with developmental stuttering generally exhibit symptoms by the age of six and about 65–85% recover from dysfluency by the age of 16 (Maguire et al., 2020).

The etiology of stuttering is likely multifactorial, including genetics, abnormal development of the basal ganglia, white matter tracts, and possibly others (Maguire et al., 2020). Twin and family studies have shown that genetics may account for about 50–80% of stuttering, with a higher concordance for stuttering in monozygotic twins than dizygotic twins and a higher risk of stuttering in those with affected first degree biological relatives (Yairi and Ambrose, 2013).

Several brain imaging studies have been conducted to elucidate the association between stuttering and certain regions of the brain. Functional magnetic resonance imaging has suggested that persistent chronic stuttering is characterized by lower activity of the left hemispheric speech areas (less active than analogous areas in the right hemisphere) (De Nil et al., 2000). For example, a large Single Photon Emission Computed Tomography (SPECT) study found that stuttering was associated with abnormally low brain activity in left-sided speech cortical areas (Pool et al., 1991). Furthermore, analysis of functional and structural brain imaging in people who stutter has indicated that brain activity in the left frontal precentral cortex is significantly lower in those who stutter than their fluent-speaking controls prior to therapy (Watkins et al., 2008). Additionally, structural differences in the left inferior frontal and premotor cortex have been reported in both children and adults with developmental stuttering, suggesting the involvement of premotor and prefrontal mechanisms in speech (Chang et al., 2011; Beal et al., 2012). A previously published meta-analysis further reported findings from diffusion tensor imaging (DTI) studies that indicated lower fractional anisotropy (FA) values in the left hemisphere of those who stutter (Neef et al., 2015). Imaging studies reveal differences in white matter function and dopamine, both of which are likely involved in stuttering pathology (Civier et al., 2013).

Compatibly, functional neuroimaging studies have suggested that left hemisphere impairments may lead to increased right hemisphere involvement in adults, suggesting a compensatory mechanism for the low functioning left hemisphere in those who have stuttered for a long period of time (Fox et al., 1996; Braun et al., 1997; Ingham et al., 2000; Samelin et al., 2000; De Nil et al., 2001; Sommer et al., 2002; Neef et al., 2018; Chang and Guenther, 2020). Functional magnetic resonance imaging has also suggested that persistent chronic stuttering is characterized by overactivation of the right frontal motor regions (Neef et al., 2018). Positron emission tomography (PET) and functional magnetic resonance imaging (fMRI) studies looking at brain functioning in people who stutter suggested higher activity in the cerebellar vermis and right anterior insular cortex during speech production, compared to their fluent-speaking controls (Fox et al., 1996, 2000; Braun et al., 1997; De Nil et al., 2000, 2003; Ingham, 2001; Neumann et al., 2003; Van Borsel et al., 2003; Watkins et al., 2008).

There also seems to be two cortical speech circuits connecting the speech cortical areas (Broca’s and Wernicke’s) that differentiate spontaneous stuttering from induced fluency (Riley et al., 1997). The inner cortical speech loop, which includes the striatum, seems to be affected in spontaneous stuttering whereas induced fluency (chorus, singing, reading out loud with another person) can bypass the inner speech loop, activating the outer speech loop as indicated by activation of the Broca’s area (Riley et al., 1997; Maguire et al., 2020). In this context, a study that utilized [^18^F] deoxyglucose (FDG) PET to measure glucose metabolism in stuttering found that stuttering was associated with abnormally low activity in the cortical speech areas and the striatum (Wu et al., 1997b). During a state of induced fluency, activity in the cortical speech areas returned to normal or high normal, but activity in the striatum of basal ganglia remained low (Wu et al., 1997b). Therefore, it was hypothesized that the basal ganglia likely play a key role in the development of stuttering. Basal ganglia are the largest subcortical structures in the forebrain and are involved in emotions, cognition, and motor processes (Alm, 2004). The striatum (putamen, caudate nucleus, and ventral striatum) is the major input nucleus, which receives excitatory projections from the cerebral cortex (Alm, 2004). The striatum is in close proximity to the globus pallidus, in which the internal part of the globus pallidus is the main output nuclei of the basal ganglia (Alm, 2004). Through the nuclei in the thalamus, it projects to the cortical areas of the frontal lobe (Alm, 2004). Higher levels of dopamine have been implicated in some people who stutter (Wu et al., 1997b; Maguire et al., 2000b).

Recently, it was proposed that the primary impairment in stuttering is a malfunctioning left hemisphere cortico-basal ganglia-thalamocortical (cortico-BG) loop involved in initiating speech motor programs (Civier et al., 2013; Chang and Guenther, 2020). There are three major areas of impairment suggested by this framework. One is an impairment in the axonal projections between the cerebral cortex, basal ganglia, and thalamus. Impaired connectivity between these regions can lead to difficulty in initiating the next sound by the basal ganglia motor loop and generating initiation and termination signals to the supplementary motor area (Civier et al., 2013; Chang and Guenther, 2020). The second area suggested to be impaired is the cerebral cortex, as seen in neurogenic stuttering where there is damage to the speech related areas in the left cortical hemisphere and in developmental stuttering where there are changes to the premotor cortex and left inferior frontal cortex (Civier et al., 2013; Chang and Guenther, 2020). Another area of impairment is the basal ganglia, evidenced by a functional magnetic resonance study, which discovered a positive correlation between stuttering severity and neural activity during speech in the striatum (Giraud et al., 2008).

In support of the basal ganglia involvement in stuttering, dopamine D2 receptor antagonists (such as haloperidol and risperidone) have been suggested to be effective in the treatment of stuttering (Maguire et al., 2020). Additionally in limited reports, medications that increase dopamine levels, such as L-dopa, have been associated with exacerbations in stuttering symptoms (Burd and Kerbeshian, 1991). Haloperidol, a first generation dopamine antagonist, has been studied in a limited manner for the treatment of stuttering with suggested positive results on efficacy. A previous study that utilized SPECT to investigate the effects of haloperidol on brain activity found that there was an increase in brain activity in speech areas, with subsequent improvement in symptoms upon haloperidol administration (Wood and Stump, 1980). Furthermore, improved fluency was associated with greater brain activity in speech areas (Wood and Stump, 1980). However, haloperidol is not a viable treatment for stuttering due to its poor tolerability and side effects (Rosenberger et al., 1976). Risperidone, which is a second generation dopamine antagonist with relatively lower risk of motor system side-effects compared to haloperidol, has been suggested in one limited, small sample size, preliminary trial, to improve fluency in adults who stutter (Maguire et al., 2002). It may also improve control over voluntary speech and involuntary tic-like movements by reducing the effect of dopaminergic projections on the left caudate nucleus, as suggested by a single-case study (Tavano et al., 2011).

In addition, stuttering has been recently postulated to be related to glial pathology (Han et al., 2019). Astrocytes, the abundant star-shape CNS glial cells, play a major role in providing neurons metabolic support, synapse formation, and synaptic function (Araque et al., 1999; Araque and Navarrete, 2010; Sofroniew and Vinters, 2010; Allen and Lyons, 2018; Marina et al., 2018). Astrocytes are heterogeneous cells with respect to their morphology, function, transcriptomes, and proteomes (Zhang and Barres, 2010; Oberheim et al., 2012; Chai et al., 2017; Miller, 2018; Sheikhbahaei et al., 2018a; Xin et al., 2019). In addition, since astrocytes can regulate activities of motor circuits and control complex behaviors (Sheikhbahaei et al., 2018b), a defect in astrocytic function can potentially affect normal functions of motor circuits controlling speech production. Interestingly, in the mouse model of stuttering, it was shown that the number of a subgroup of astrocytes [identified by Glial fibrillary acidic protein (GFAP)] in the corpus callosum was reduced (Han et al., 2019). It is also accepted that human astrocytes are functionally and morphologically more complex than those in rodents (Oberheim et al., 2006, 2009, 2012; Vasile et al., 2017). Although more experiments are required to define the functional significance of this reduction in number of astroglia cells, data suggest that astrocytes might play a critical role in the development of stuttering. Involvement of astrocytes in motor control disorders is not without a precedent as it has been shown that they have a critical role in the pathogenesis of Parkinson’s disease, amyotrophic lateral sclerosis (ALS), Tourette’s Syndrome, and others (van Passel et al., 2001; Nagai et al., 2007; Chen et al., 2009; Rappold and Tieu, 2010; Haidet-Phillips et al., 2011; Meyer et al., 2014; de Leeuw et al., 2015; Booth et al., 2017; Yamanaka and Komine, 2018; Yun et al., 2018; Cressatti et al., 2019).

The purpose of the current study was to examine the possible effects of risperidone on regional brain metabolism, in stuttering. This preliminary study was devised using neuroimaging data from a previous study conducted in the year 2000 in order to test the hypothesis that risperidone would increase striatal and Broca’s area function, which may correlate its association with the presence of stuttering symptoms. We propose that the therapeutic effect of risperidone might be, in part, due to increased metabolism of striatal astrocytes.

## Methods

### Participants

The participants were a subset from a previous study (Maguire et al., 2000a). While 16 subjects (12 males and four females) were enrolled in the study only 10 subjects (eight males and two females) gave consent to undergo a PET scan. The randomized, double-blind, placebo-controlled study examined stuttering in 10 English speaking, adult participants (20–74 years old), with no significant differences between the groups in age, gender, and stuttering severity at baseline. In order to account for possible stress associated with the solo reading task, only those who had completed their senior year of high school were included. Furthermore, all subjects were right-handed and right eye, right foot dominant. Additionally, they had the developmental rather than the acquired form of stuttering, with symptoms present before 6 years old. They also had a minimum score of 15 on the SSI-3 (Riley, 1994), and of the total syllables spoken, they had a minimum severity of at least 3% of syllables stuttered. We assessed stuttering severity by asking the patient to describe a non-emotionally laden event and to read from standardized passages with 500 syllables (Maguire et al., 2000a). The recorded videos and audio tapes were analyzed to determine the stuttering frequency (%SS), duration of stuttering, time spent stuttering in relation to the total time speaking (%TS), and the overall stuttering severity score (Maguire et al., 2000a). None were receiving speech therapy for at least 6 months before the study and for the duration of the study. All had no prior history of pharmacological treatment of stuttering. Subjects were excluded if they had major medical issues, prior history of treatment with antipsychotics, or were using psychoactive medications or drugs of abuse. Informed consent was obtained prior to the study in accordance with the institutional review board at the University of California, Irvine.

### Procedure

Subjects who satisfied the criteria had their stuttering severity rated twice during a baseline period of 2–4 weeks (Maguire et al., 2000a). Then they were randomly assigned to receive 6 weeks of treatment with either risperidone or identically appearing placebo pills. Those who were taking risperidone were started on 0.5 mg daily at night. The dose was increased by 0.5 mg/day every four or more days as tolerated up to a maximum of 2.0 mg/day. During the 6 weeks, compliance with the medication, stuttering severity, side effects, tolerability were rated every 2 weeks. Stuttering severity was also assessed and recorded after risperidone or placebo treatment. Medication compliance was evaluated by looking at participants’ daily written record, and confirmed by comparing the number of pills dispensed to the number of pills returned.

Subjects received two FDG PET scans (the measured resolution of the scanner was 7.6 mm in plane and 10.9 mm in the z-dimension), one prior to randomized placebo/medication treatment, and one at 6 weeks while still receiving the double-blind therapy. FDG was used as the marker in order to visualize the regional glucose metabolic processes in the brain activated during speech (Wu et al., 1995). To visualize glucose metabolism, subjects read aloud an emotionally unburdened article to another individual for 30 min before entering the scanner. Thirty minutes of reading aloud was chosen for the standard of allowing the uptake of FDG for imaging visualization.

Subjects lay quietly in a supine position with their eyes open for the entire duration of the PET scan, which was 90 min – making the total procedure time 2 h. The study was limited to just two scans because of the accumulative radiation exposure. The treatment blind (risperidone or placebo) was broken after all the subject scans were obtained and the data was analyzed. With such, we found that there were an equal number of subjects in each group randomized (five on risperidone, five on placebo). Furthermore, we observed differences in the maximum response to risperidone, with some exhibiting maximum response at 2 weeks when subjects were receiving 0.5 mg/day and some showing maximum response at 6 weeks. In order to account for the differing dose related response, subjects in each group were ranked by stuttering severity and each subject in the risperidone group was matched with an equally ranked subject placebo group. Therefore, those in the risperidone group who showed maximal response at the low doses (2 weeks) were compared with those who showed a similar response in the placebo group. The same applied to those who showed maximal response at high doses (6 weeks).

### Data Analysis

Positron emission tomography images were reconstructed using standard calculated smoothing filter and attenuation correction methods described elsewhere (Sokoloff et al., 1977; Friston et al., 1991a,b). The images were then anatomically normalized using standardized MRI based atlas. Anatomical localization was accomplished through the concordance of Talairach and Tournoux coordinates (Friston et al., 1991b) and regions of interest (ROI) determined by a probabilistic brain atlas as described elsewhere (Potkin et al., 2003). The probability for a given profile of contiguous connected clusters exceeding the threshold of *p* < 0.05 was calculated by resampling-based image cluster analysis (Wu et al., 1997a). All significant voxels with cluster sizes less than the calculated threshold cluster size (37 voxels) were not analyzed. Paired *t*-tests were performed for stuttering subjects comparing their pre-treatment condition with their post-treatment condition. Areas of increased or decreased metabolism were identified using an overlay of Brodmann defined regions from MRIs. Only significant areas (*t* > 3.2) were reported.

## Results

**FIGURE 1:**
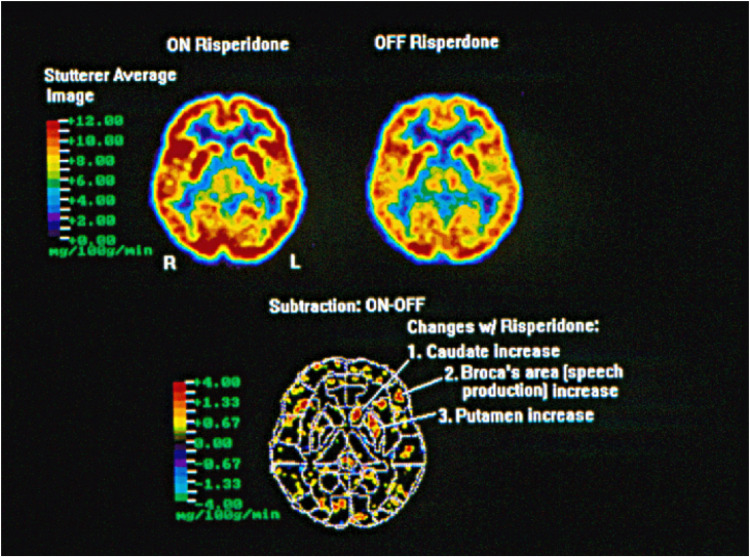
Within group analysis FDG PET scan of five adult subjects who stutter scanned before treatment (off risperidone) and after treatment for 6 weeks (on risperidone) taken after a 30-min solo reading aloud task. Subjects on risperidone received 0.5–2.0 mg based on tolerability. The acquisition of the FDG during the reading aloud task was 30 min and the scan acquisition time was 90 min. Images were anatomically normalized using the coordinate system of the Talairach atlas (Friston et al., 1991b). Areas of increased (red) or decreased (blue) metabolism were identified using an overlay of Brodmann defined regions from MRIs (Wu et al., 1995). All regions of the brain were examined, with threshold differences of *p* < 0.05 identified for the caudate, putamen, and Broca’s area (the mg/100 g/min scale represents the uptake rate of the isotope).

Treatment efficacy, determined by %SS, was significantly higher in the risperidone treatment group compared to the placebo group (*p* = 0.025) for the original study sample (Maguire et al., 2000a). Additionally, the mean reductions in the overall group for %SS (from 9.6 ± 8.2 to 4.7 ± 4.6), duration (from 4.5 ± 5.1 to 3.2 ± 3.8),%TS (from 28.0 ± 23.8 to 16.7 ± 19.4), and SSI-3 (from 25.3 ± 8.6 to 17.5 ± 9.8) of the risperidone group were greater than those of the placebo group (from 7.0 ± 3.7 to 5.1 ± 3.0, from 3.3 ± 3.0 to 2.8 ± 3.3, from 20.4 ± 13.9 to 16.4 ± 13.6, and from 24.0 ± 9.9 to 20.5 ± 8.6, respectively), with the risperidone group displaying statistically significant changes in SS% (*p* < 0.01), %TS (*p* < 0.01), and SSI-3 (*p* < 0.0001) (Maguire et al., 2000a). Overall, risperidone was well tolerated, with the most common side effect being sedation and one reported case of galactorrhea and amenorrhea, which resolved 2 months after the medication was discontinued (Maguire et al., 2000a). FDG PET scans taken before randomization revealed similarities in brain activation among placebo and active treatment groups. Scans of subjects on placebo after 6 weeks showed no difference from baseline. Alternatively, the subjects receiving risperidone (“on risperidone”) after 6 weeks exhibited increased activity from baseline (“off risperidone”) in the left caudate, putamen, and Broca’s area (*p* < 0.05). See Figure 1 below.

## Discussion

Data presented in this study suggest that risperidone treatment is associated with increased activity of the striatum and Broca’s area in persons who stutter. The subtraction image generated from the individual FDG PET scans demonstrate enhanced brain activity in risperidone-treated subjects in three particular regions: the left caudate and putamen (components of the striatum in the basal ganglia) and the Broca’s area. A previous study demonstrated persistent left caudate hypometabolism, as well as reversible left language circuit hypometabolism through induced fluency (Wu et al., 1995). This study further supports the hypothesis that the basal ganglia are central to stuttering and as such may serve as a possible biologic marker for stuttering.

While we only observed changes in the left hemisphere, this does not imply that there are no changes in the right hemisphere. The right hemisphere was also imaged in our study and may play a role in stuttering and stuttering recovery, but the medication effect observed in this study in right-handed individuals was solely related to the left hemisphere. Recent findings have suggested increased right hemisphere involvement in those with impaired performance of the left hemisphere cortical network for speech, with the right hemisphere compensating for abnormalities in the white matter tract of the left hemisphere (Riley et al., 1997). Additionally, there is evidence to suggest that certain forms of speech therapy may impact the right hemisphere. For example, in fluency-inducing therapy, successful treatment was associated with the shift from right hemisphere cortical activity toward left lateralized frontal activation (De Nil et al., 2003; Neumann et al., 2005).

Although the pathology of stuttering is not fully understood, stuttering has recently been postulated to be related to glial pathology (Han et al., 2019). Similar to neurons, astrocytes in different brain regions also express functional dopamine receptors (D1 and D2) and dopamine transports (Pelton et al., 1981; Jennings and Rusakov, 2016). Application of dopamine triggers profound intracellular changes in murine astrocytes (Requardt et al., 2012). Therefore, medications affecting D1/D2 can have subtle effects on astrocytes as well. In fact, administration of risperidone increased astrocyte activity (measured by GFAP reactivity or glutamine synthetase) in rats, monkeys, and humans (Toro et al., 2006; Bernstein et al., 2014; Fernandez et al., 2019). In rhesus monkeys, the effect of risperidone was restricted to the striatum as histological staining showed reversible increase in staining in the cell bodies as well as processes of putamen astrocytes (Fernandez et al., 2019). Another dopamine receptor blocker, haloperidol, was shown to block D2 receptors on astrocytes. Chronic administration of this medication increased astrocyte metabolic activity in rats (Konopaske et al., 2013). By extrapolation, it is reasonable that risperidone might have a similar effect on astrocytes. Therefore, the increased metabolic activities of the putamen and Broca’s area that we observed in our study could be, in part, due to the activation of astrocytes; when activated, basal ganglia astrocytes can inhibit dopaminergic neurons (Xin et al., 2019). Together, our data further strengthen the hypothesis that astrocytes may play a major role in the development of stuttering. Given that both white matter function and dopamine activity have been implicated in stuttering, a possible line of further research may be the investigation of risperidone and related medications on astrocyte activity and the possible relation to white matter integrity (Szeszko et al., 2014; Dutta et al., 2018).

One of the initial PET studies used intravenously administered oxygen-15 labeled water [(Ingham et al., 2000) O-water] to measure regional cerebral blood flow (Verger and Guedj, 2018). This was a sensitive method to quantify regional brain activation during various tasks and was useful in mapping brain activation patterns in cognitive tasks (Verger and Guedj, 2018). The PET imaging studies were followed by magnetic resonance imaging (MRI) which allowed for the study of functional connectivity through serial imaging of the brain during a resting state or task dependent activation (Verger and Guedj, 2018). Due to several limitations in these earlier PET studies, fMRI has become the functional imaging modality of choice in stuttering due to the absence of radiation exposure and enhanced resolution. However, some advantages of FDG PET over fMRI still exist. fMRI is susceptible to ferromagnetic artifacts, which makes neuroimaging a challenge due to a high prevalence of treatment with implantable devices that are incompatible with magnetic resonance (Verger and Guedj, 2018). Fortunately, ferromagnetic implants in subjects who stutter tend to be relatively rare, especially in children. FDG PET should seldom, if ever, be considered as imaging modality in children and other susceptible populations because of the radiation exposure. However, with fMRI, movement artifact from talking or orofacial movements, as well as the gradient coils, can produce noise that negatively impacts listening to auditory inputs (Verger and Guedj, 2018). Compared to fMRI, there is no auditory effect on subjects during PET imaging.

The limitations of our study include a small sample size and a relatively limited imaging technique that involves radiation exposure. Future studies should employ less invasive imaging techniques at higher resolution. Furthermore, our data were obtained at the author’s prior institution and more specific demographic data regarding the subjects were not maintained for reference in this article. Another limitation is that we utilized a flexible dose design for the study. Given the preliminary nature of the study, optimal dosing of risperidone in stuttering was based on efficacy and adverse events, as the maximum tolerable and effective dose of risperidone for stuttering were not available. Therefore, a flexible dosage design was utilized to gather understanding regarding the potential target dose for stuttering. Since risperidone is metabolized by the cytochrome P450 2D6 system, significant genetic variability exists, which may partly explain the dosing differences seen in this trial. For future studies, employing a more rigorous dosing design including fixed dose analyses in a larger sample size is required.

In summary, the preliminary findings from this study suggest larger studies involving pharmacologic interventions in stuttering may possibly predict which patients may respond to therapy and which will not. More definitive studies may yield further insights into the neurophysiology of stuttering. Integrating functional neuroimaging techniques into the treatment of stuttering may eventually prove to guide interventions and predict responders and non-responders to various forms of therapies. Future treatment intervention studies may employ functional neuroimaging to investigate the potential changes in the cortico-BG loop. Moving forward, as the potential for pharmacological treatment in stuttering grows, it is unlikely that all stuttering subjects will show a uniform response. Therefore, imaging may prove to be a useful tool in guiding future clinicians as to which therapies may be personalized for each subject, optimizing response and minimizing undue risks. Moreover, research in animal models of stuttering could be critical in improving our understanding of glial role in stuttering, studying the mechanism of medication therapies, and developing cell-specific therapies to target striatal astrocytes.

## Data Availability Statement

The original contributions presented in the study are included in the article/supplementary material. Further inquiries can be directed to the corresponding author.

## Ethics Statement

The studies involving human participants were reviewed and approved by UC Irvine. The patients/participants provided their written informed consent to participate in this study.

## Author Contributions

GM designed the study, captured and analyzed the data, and participated in the manuscript writing. BY participated in writing the manuscript and conducted the background literature review. SS revised the article critically for important intellectual content. All authors contributed to the article and approved the submitted version.

## Conflict of Interest

GM in the past received a research grant from Janssen which supported in part the randomized clinical trial linked to this imaging study. However, the imaging study was funded purely from a philanthropic donation from RG Kirkup. At this time, the medication, risperidone is generic and Janssen no longer has exclusivity on the sales of this compound. GM currently has research grants from Teva and Emalex. GM also serves on the advisory board of Vivera Pharmaceuticals. In the past year, but no longer continuing, GM also received speaking honoraria from Otsuka, Sunovion, Janssen, Intracellular, Takeda, and Eisai. The remaining authors declare that the research was conducted in the absence of any commercial or financial relationships that could be construed as a potential conflict of interest.
